# Does Cycling Training Reduce Quality of Functional Movement Motor Patterns and Dynamic Postural Control in Adolescent Cyclists? A Pilot Study

**DOI:** 10.3390/ijerph191912109

**Published:** 2022-09-24

**Authors:** Bartosz Zając, Anna Mika, Paulina Katarzyna Gaj, Tadeusz Ambroży

**Affiliations:** 1 Laboratory of Functional Diagnostics, Central Scientific and Research Laboratory, University of Physical Education, 31-571 Kraków, Poland; 2 Institute of Clinical Rehabilitation, University of Physical Education, 31-571 Kraków, Poland; 3 Cracow Academy of Track-and-Field Sports, 31-511 Kraków, Poland; 4 Institute of Sport Science, University of Physical Education, 31-571 Kraków, Poland

**Keywords:** FMS test, Y-balance test, cycling training, overuse injury

## Abstract

The aim of this study was to assess whether cycling training may influence quality of functional movement patterns and dynamic postural control. We also sought to determine if the Functional Movement Screen and Lower Quarter Y-balance tests could be predictive of injury risk among adolescent road cyclists. Twenty-three male road cyclists, aged 15–18 years, were involved in the study. Quality of functional movement patterns was assessed using the Functional Movement Screen test (FMS). Dynamic postural control was evaluated using the Lower Quarter Y-balance test (YBT-LQ). Information on injury occurrence was collected through a retrospective survey. The results showed the highest percentage of scores equalling 0 and 1 (>30% in total) in two FMS component tests: the hurdle step and trunk stability push-up. The results also demonstrated a low injury predictive value of the Functional Movement Screen (cut-off <14/21 composite score) and the Lower Quarter Y-balance test (cut-off <94% composite score and >4 cm reach distance asymmetry) in adolescent road cyclists. The most important information obtained from this study is that youth road cyclists may have functional deficits within the lumbo-pelvic-hip complex and the trunk, while neither the FMS nor the YBT-LQ test are not recommended for injury risk screening in cyclists.

## 1. Introduction

Non-traumatic (overuse) injuries are a real problem among professional and amateur road cyclists [1]. Clarsen et al. [2] showed that 94% of world class cyclists experience injuries during a 1-year period, where symptoms of both lower back pain and anterior knee pain were common, with an annual prevalence of 58% and 36%, respectively. Barrios et al. [3], in professional road cyclists, reported about five non-traumatic injuries per 100,000 covered km (mainly tendinitis of the patellar and Achilles tendon). Furthermore, during the year prior to the survey, a high level of pain and overuse injuries were observed in amateur cyclists, reaching 85 [4] and 88% [5]. What is of great significance is that each injury resulted in a loss of training time and socio-economic costs, and impeded competitive success [6,7,8,9]. Therefore, detection of specific risk factors for cyclists is crucial in decreasing the rate of sport-related musculoskeletal injuries [10,11].

Road cycling is one of the most demanding endurance sports for the body. World-class road cyclists typically train a mean of 20 h per week and cover 600 km per week [12]. During this time, they assume a characteristic cycling position to reduce aerodynamic drag [13,14,15], which requires extreme horizontal trunk flattening and hip flexion, achieved, in part, through anterior pelvic tilt [16]. In some studies, it has been shown that cyclists sitting on a bicycle modified the lumbar lordosis curve to kyphosis [17,18]. This situation may cause posture abnormalities such as increased standing thoracic kyphosis [19] or greater anterior pelvic tilt in a seated position [16] compared to sedentary individuals. Furthermore, San Emeterio et al. [20] found that in elite female cyclists, intense cycling training induced significant alterations in lumbopelvic movement. The specificity of training also includes mainly endurance exercise and general strengthening exercise, usually not focused on lumbo-pelvic stability and on hip mobility [21,22,23]. However, there are not many studies in which the impact of road cycling training on neuromuscular control and movement quality is addressed using movement-competency base tests such as the Functional Movement Screen (FMS) and/or the Lower Quarter Y-balance test (YBT-LQ). Moreover, the sensitivity and specificity of the FMS and YBT-LQ as tools for injury prediction in the population of cyclists has not been reported.

FMS is a screening test that was developed with the goal of identifying deficits in movements that may predispose an otherwise healthy person to injuries during physical activity [24,25]. FMS allows the assessment of stability and mobility within the kinetic chain of full body movement, the identification of painful patterns and body asymmetries, and the recognition of overall poor-quality movement patterns [21,24,25]. The Functional Movement Screen is inexpensive, easy to use, and shows acceptable intra- and inter-rater reliability [26,27]. However, like any tools, the FMS has limitations, i.e., task-specific evaluation criteria and high intra- and inter-individual variability in movement coordination and control [28], as well as equivocal injury predictive value of the composite score [29,30,31,32,33,34,35,36].

YBT-LQ is extensively used for injury risk identification [37], return to sport testing [38], and pre–post intervention measurement [39,40,41]. YBT-LQ aids the assessment of dynamic neuromuscular control and lower-extremity functional flexibility. The Lower Quarter Y-balance test, like FMS, is inexpensive, easy to use, and shows good intra- and inter-rater reliability [42]. However, the relationship between composite score, reach distance (raw and normalized), reach distance asymmetry, and injury risk remains unclear [43,44,45,46,47,48,49].

We have hypothesized that because of long-time exposure to specific cycling positions, in combination with a high level of physical effort, road cyclists may be prone to specific neuromuscular alternations. Therefore, the aim of this study was to assess whether cycling training influences quality of functional movement patterns and dynamic postural control. We also sought to determine if the Functional Movement Screen and Lower Quarter Y-balance tests may be predictive in injury risk assessment among adolescent road cyclists.

## 2. Materials and Methods

### 2.1. Participants

The study involved 23 male road cyclists, aged 15–18 years, recruited from students of the Sport Championship School in Świdnica, Poland. The inclusion criteria were: (i) minimum 1-year training experience; (ii) obtaining a sports result at the level of at least the second sports class (according to the standards established by the Polish Cycling Federation) in the 12 months preceding the study; (iii) average training volume above 8 h per week in the last 3 weeks before the study, registered with a sport-tester; (iv) having a current certificate from a sports medicine doctor regarding the ability to practice road cycling. The exclusion criteria were acute injuries that made it impossible to perform the tests. The characteristics of the study population are presented in Table 1.

### 2.2. Experimental Design

All measurements were performed by an experienced researcher during one visit at the beginning of the preparatory period. The Functional Movement Screen (FMS) and Lower Quarter Y-balance test (YBT-LQ) were performed with a 5 min break in between and in a random order. Additionally, at baseline, anthropometric measurements and information about training experience were collected. All measurements were performed between 8:00 a.m. and 4:00 p.m. in a sports hall at an ambient temperature of 20 + 1 °C. The participants were asked by e-mail not to perform intensive training sessions within 48 h prior to testing. Eighteen weeks after measurements, participants were asked to fill in a retrospective survey on the prevalence of injuries occurring since the time of the baseline tests.

### 2.3. Anthropometric Measurements

Body height was measured via an anthropometer (Metrisis, Thessaloniki, Greece). The length of the lower limbs was measured in supine position (anterosuperior iliac spine to centre of the ipsilateral medial malleolus) via a measuring tape (TK Gruppe Timo Klingler, Shenzhen, China) [50]. Body mass and fat mass were determined with a body composition multi-frequency octopolar analyser (Tanita MC 780 MA, Tokyo, Japan) using the method of electrical bioimpedance in sport mode [51]. Before measurements, the feet and hands of the subject and the analyser were cleaned and degreased.

### 2.4. Functional Movement Screen Test (FMS)

The purpose of the FMS test was to assess fundamental movement patterns. Participants completed seven parts of the test (deep squat, hurdle step, in-line lunge, shoulder mobility, active straight leg raise, trunk stability push-up, and quadruped rotary stability) using the FMS kit (Perform Better, Cranston, RI, USA) in accordance with the methodology described by Cook et al. [52,53]. Participants were given verbal instructions for task performance and were allowed three attempts for each task (maximal score was registered). Then, the scores of all seven test parts were summed, resulting in a composite score (0–21 points). Five of the seven test parts assessed asymmetry by bilateral measurements. If discrepancies existed between the left and right sides, the score for the worse side was registered. Each test component was scored on an ordinal scale (0–3 points) based on quality of movement, with three being the maximum score. A score of 2 indicated that the participants required some type of compensation or were unable to complete the entire movement. A score of 1 was given if the individual was unable to remain in the movement position throughout the movement, lost balance during the test, or did not meet the minimum criteria to score a 2. Pain during any of the FMS component tests indicated a score of 0. In addition to the seven component tests, the FMS includes three clearing tests for pain detection (shoulder internal rotation and abduction with the hand placed on the opposite shoulder, lumbar extension performed in prone press-up position, and end-range lumbar flexion in quadruped). Pain on a clearing test resulted in a score of 0 for the shoulder mobility, trunk stability push-up, or rotatory stability tests, respectively. Details on scoring for each of the component tests are provided in the works by Teyhen et al. [54] and Cook et al. [52,53]. FMS test reliability for the ICC inter-rater ranged from 0.87 to 0.89, and for the ICC intra-rater, the range was from 0.81 to 0.91 [55,56].

### 2.5. Lower Quarter Y-Balance Test (YBT-LQ)

The YBT-LQ test, which was applied to evaluate quality of dynamic postural control, was performed using the YBT kit (Move2Perform, Evansville, IN, USA). The participants pushed the reach indicator blocks with one foot as far as possible in three directions—anterior (A), posteromedial (PM), and posterolateral (PL)—while standing on the contralateral leg on a central platform with hands on the pelvis. Each participant was allowed six practice trials in each direction and then performed three test trials in each direction. The reach distance was recorded as the point where the participant pushed the reach indicator block closest to the central platform to the nearest 1 cm. The testing order was as follows: three trials standing on the right foot reaching in the anterior direction (right anterior reach) followed by three trials standing on the left foot reaching in the anterior direction. This procedure was repeated for the posteromedial and the posterolateral reach directions. The trail was discarded and repeated if the subject: (i) failed to maintain a unilateral stance on the platform (e.g., touched down to the floor with the reaching foot or fell off the stance platform); (ii) failed to maintain the reach foot in contact with the reach indicator on the target area while in motion (e.g., kicked the reach indicator); (iii) used the reach indicator for stance support (e.g., placed foot on top of reach indicator); (iv) failed to return the reach foot to the starting position under control; (v) broke stance foot–heel from central platform. The average of three successful test trails for each reach direction was used for data analysis. Normalised reach distance and composite scores (CS) were calculated according to the formula proposed by Bulow et al. [57]:

(1) $$normalised\;reach\;distance=\frac{excursion\;distance}{leg\;length}\times 100\%$$

(2) $$CS=\frac{RD\left(anterior\right)+RD\left(posteromedial\right)+RD\left(posterolateral\right)}{3\times leg\;length}\times 100\%$$

The reported reliability of the YBT-LQ was 0.85–0.91 for the ICC intra-rater and 0.85–0.93 for ICC inter-rater [45,58].

### 2.6. Overuse Injury Survey

A retrospective survey was used to collect information about overuse injuries in five anatomical locations (knee, hip, back, neck, and other body areas) that occurred during the previous 18 weeks. Five questions included in Table 2 were repeated for each location. Before the survey, subjects were informed and familiarised with the definition of overuse injury (any pain or discomfort that was not directly associated with a traumatic event and was different from the normal pain associated with competitive cycling). Any physical complaint sustained by cyclists that resulted in training volume reduction was adopted as an injury criterion for sensitivity, specificity, and odds ratio calculation [59].

### 2.7. Statistical Analyses

Statistical analysis was performed using SPSS Statistics 26 (IBM, Armonk, NY, USA). Differences between the right and left sides (for Y-balance test reach distances and composite score) were evaluated with the *t*-test for independent samples or the Mann–Whitney’s U test, depending on the assessment of normal distribution. The normality of distribution was examined via the Shapiro–Wilk test. The probability of Type I error below 0.05 was adopted as the level of significance. The median as well as the first and third quartiles were used to present the results of the study. Sensitivity, specificity, odds ratio, and 95% confidence interval were calculated according to the proposal by Altman [60] and Altman et al. [61].

## 3. Results

### 3.1. Functional Movement Screen Test (FMS)

The FMS test indicated a high percentage (in total >30% all cases) of scores totalling 0 and 1 in hurdle step and trunk stability push-up tests compared to the remaining tests (Table 3). The median as well as first and third quartiles of FMS composite score reached a value of 15 (13–17).

### 3.2. Lower Quarter Y-Balance Test (LQ-YBT)

There were no demonstrated statistically significant differences between the right and left sides in the YBT-LQ composite score (Table 4). There were also no statistically significant differences between the right and left sides with regard to anterior, posterolateral, posteromedial raw (cm), and normalized (% leg length) reach distances (Table 5).

### 3.3. Overuse Injury Survey

The result of the survey showed overuse injuries in six cyclists (31.6% of valid cases). Half of them reported injuries in more than one location. A total of 12 injuries were recorded, with most of them localized in the knee (26.3% of valid cases) and back (21.1% of valid cases). All reported knee injuries lasted between 4 and 7 days. All cyclists who reported knee problems had to reduce the training load and experienced pain, at least to a moderate extent. Half of the cases regarding knee injuries affected performance. The duration of the back problems varied considerably. Half of cases with back problems did not require a reduction of training load. The majority of back injury cases experienced at least moderate pain and were forced to reduce training loads. Detailed numerical data concerning the location and severity of injury are presented in Table 6.

### 3.4. Injury Prediction Value of FMS and YBT-LQ

Indicators of injury risk predictive value (sensitivity, specificity. and odds ratio) of the Functional Movement Screen and Y-balance tests are presented in Table 7.

## 4. Discussion

The results of the current study showed that the values of the FMS composite score (FMS_CS_) as well as those of the YBT-LQ test (YBT-LQ_CS_) in youth road cyclists were similar to those observed by other authors in adolescent populations. However, the lowest scores were noted in hurdle step and in the trunk stability push-up for the FMS test, which may suggest the existence of core stability deficits. Moreover, despite good results of the FMS_CS_ and YBT-LQ tests, the evaluated adolescent cyclists suffered from overuse injuries during the 18-week period after conducting the assessment.

In our study, we noted a high percentage of 0 and 1 scores (in total, >30% of all cases) in two FMS component tests (hurdle step and trunk stability push-up) compared to other adolescent populations [62,63,64]. The hurdle step task is mainly used to assess unilateral hip extensor mobility and postural control [24]. Given the fact that for the evaluated cyclists, the results of YBT-LQ test did not indicate abnormalities in dynamic postural control, it can be suggested that cycling training may predispose to limitations in hip extension mobility. The trunk stability push-up task, in turn, is applied to evaluate core stability in the sagittal plane during symmetric upper limb movement [25] and upper body strength [65]. The alterations presented in this test may be attributed to poor stability of the trunk stabilisers [25]. Therefore, it may be probable that spending significant time in cycling position, which requires extreme trunk horizontal flattening and hip flexion achieved through excessive anterior pelvis tilt [16] in combination with a high level of physical effort [20], may overload tissues and lead to some deficits in the mobility (mainly in hip extension) and stability of the lumbo-pelvis complex and/or trunk. Furthermore, the potential reason for functional movement deficits observed in evaluated cyclists may be related to overall training specificity, which is mainly focused on endurance exercises and on general strength training [21,22,23]. However, specific functional exercises focused on lumbo-pelvic complex mobility and stability are very rarely performed by cyclists. Because cyclists spend most of their training time on a bike, much less time is devoted to functional exercises, which may negatively affect core musculature performance during stability tasks off a bike [23]. During the 18 weeks following evaluation, despite good results on the FMS_CS_ and YBT-LQ, more than 30% of studied group experienced some overuse injuries, mainly located in the knees and back. Therefore, we may suggest that those two parts of the FMS test (hurdle step and trunk stability push-up) may be indicative of weak points of the body in young cyclists that may be vulnerable to overload.

Abraham et al. [62] showed that the mean FMS_CS_ value in 10–17-year-old school students of both genders was 14.59 + 2.48. Similarly, Anderson et al. [66], in 31 healthy male secondary school athletes (age 16.0 + 1.1 years), reported a mean FMS_CS_ value of 15.3 + 2.1. Rannama et al. [67] and Rannama et al. [68] demonstrated FMS_CS_ values totalling 14.7 + 1.6 and 14.13 + 1.80 in male road cyclists (age 19.2 + 2.3 and 18.5 + 2.1 years, respectively). Only Pfeifer et al. [69] reported a slightly lower mean and larger standard deviation of FMS_CS_, that is, 12.62 + 3.06 (mode 14), in 63 male high-school students (age: 15.87 + 1.44 years). These FMS_CS_ values are similar to those obtained in our study (median: 15, 1st-3rd quartiles: 13–17). However, we should be careful with the interpretation of the FMS_CS_ result, because it is a combination of multiple subtests used to assess different movement qualities (i.e., mobility, strength, balance, etc.) in different body regions. Moreover, the weaknesses of this test are the task-specific criteria and high inter-individual variability in movement coordination and control, whereby a score of 2 points for a given test can be obtained in several different ways. Although FMS_CS_ may be similar between the evaluated athletes, these scores could have been obtained with different individual movement patterns [28,70].

The low sensitivity (SP) 0.33 (95% CI 0.04–0.78), specificity (SE) 0.62 (95% CI: 0.32–0.86), and odds ratio (OR) 0.80 (95% CI: 0.10–6.10) observed in our study indicated poor ability of FMS_CS_ < 14/21 to predict injury risk in youth road cyclists. For other studies in physically active populations, equivocal results were provided. Some authors underlined that poor performance on the FMS_CS_ may reflect a history of injury rather than predict future injury risk [70]. In the majority of meta-analysis reviews, the strength of association between FMS_CS_ ≤ 14/21 and subsequent injury is not supported, nor is its use as an injury prediction tool [71,72,73,74]. Dorrel et al. [71] showed an SE of 0.24 (95% CI: 0.15–0.36) and SP 0.85 (95% CI: 0.77–0.91) in active adult populations. Moore et al. [72], in junior athletes, demonstrated an SE totalling 0.50 (95% CI: 0.43–0.57), SP of 0.59 (95% CI: 0.44–0.72), and OR equalling 1.16 (95% CI: 0.72–1.87), and showed that the prediction value of FMS_CS_ < 14/21, which may vary depending on population, injury mechanism, and injury definition. In male military personnel, Moran et al. [73] reported ‘strong’ evidence that the strength of association between composite score (cut-point ≤ 14/21) and subsequent injury was ‘small’. Beardsley et al. [74] even suggested that the probable predictive criterion-referenced validity value of the FMS_CS_ ≤ 14/21 may be only marginally better than a coin toss for identifying individuals who are genuinely at risk of injury. By contrast, in a review by Bonazza et al. [75] including nine prospective cohort studies, it was reported that participants (athlete, fire-fighter, and military populations) who scored ≤ 14 on the FMS have greater than two-fold—OR of 2.74 (95% CI: 1.70–4.43)—the odds of sustaining a musculoskeletal injury than those with scores > 14 (but the authors of this review ignored heterogeneity in the study population type, follow-up time, and injury definition [72]). Low injury risk predictive value of the FMS test may be due to its low construct validity. The FMS test allows the identification of compensatory, painful, and asymmetric movement patterns in tasks, which are often different from those performed in specified sports disciplines. Thus, it is equivocal how the identification of such compensatory movement patterns may be significantly linked to the prediction of sport-related injury. Furthermore, it is also worth highlighting that when using FMS_CS_, the cut-off value should be adjusted to subject age, gender, experience, and level of competition. Therefore, FMS_CS_ should be used with caution as a criterion differentiating athletes with high and low risk of injury.

The YBT-LQ composite score (YBT-LQ_CS_) reported in our study did not differ from values observed in adolescent athletes by other authors [76,77,78]. Schwiertz et al. [76], in male volunteers aged 14–15 years, observed a 97.0% level of YBT-LQ_CS_ composition score for the right leg and 98.1% for the left one. The higher values were reported in the 16–17 age group: 101.5%, and 102.8% for the right and left sides, respectively. Similarly, Alhusaini et al. [77], in healthy male children aged 12 to 15 years, demonstrated 94.13 + 8.83% for the right side and 93.68 + 8.79% for the left side. Such a value of YBT composition score (93.3 + 9.6%) was also noted by O’Connor et al. [78] in adolescent Gaelic footballers and hurlers. In our study, no asymmetry in composite score or reach distance was observed, suggesting that cycling training probably did not adversely affect the dynamic postural control of the young cyclists under evaluation.

In our study, performance on the YBT-LQ test (composite score < 94%, and reach distance asymmetry >4 cm) showed low injury prediction value in the evaluated cyclists. Moreover, in the literature, ambiguous observations have been noted. Butler et al. [49] reported an odds ratio of 3.5 (95% CI 2.4–5.3) when using a composite cut-off of 89.6% in football players. Wright et al. [46] and Brumitt et al. [45] utilised different composite cut-offs for athletic teams, ranging between 89 and 94%, all of which yielded non-significant likelihood ratios (ranges: 0.55–1.32 and 0.50–1.70, respectively). Smith et al. [43] utilised 4-cm anterior reach distance asymmetry and found a relationship with future injury risk, reporting an odds ratio of 2.20 (95% CI 1.09–4.46). Vaulerin et al. [79] found that an asymmetry of >2 cm was predictive of ankle sprains. Šiupšinskas et al. [80] reported only limb difference scores and did not find an association with injury in elite female basketball players. Gonell et al. [81] indicated an odds ratio of 3.86 (95% CI 1.46–10.95) for male soccer players with a posteromedial asymmetry of 4 cm or greater. Lehr et al. [82] used population specific cut-points for various sports. The researchers found that injury predictive values of the YBT-LQ were increased when multiple risk factors were combined. The authors applied age, sex, and sport-specific risk cut-points to place athletes in risk categories. These cut points were based on previously published injury prediction studies and normative databases. Thus, it seems important to include age, sex, and sport cut-points for injury risk identification.

The limitation of this study is the fact that in the research, a homogeneous (in terms of age and sex) and relatively small group of road cyclists was included; thus, the results cannot be generalised to a broader population. Moreover, the study population included athletes with a wide range training experience, which should be taken into account, while training experience was previously reported as injury factor [83].

## 5. Conclusions

The most important information from this study is that youth road cyclists may have functional deficits within the lumbo-pelvic-hip complex and the trunk. Moreover, neither the FMS test nor the YBT-LQ test are not recommended for injury risk screening in adolescent cyclists. Future research is needed to develop specific tests for cyclists and specific cut-points for FMS and YBT-LQ composite scores to more accurately determine future injury risk.

## Figures and Tables

**Table 1:** Characteristics of studied population.

	Median (Q1–Q3)	Minimum	Maximum	CQV (%)
Age [year]	16.0 (15.0–17.0)	15.0	17.0	6.3
TE [year]	5.0 (3.0–6.0)	1.0	8.0	30.0
Age in TE groups [year]				
1–4 year, *n* = 9	15.0 (15.0–16.0)	15.0	17.0	3.2
5–8 year, *n* = 16	16.5 (16.0–17.0)	15.0	17.0	3.0
Body height [cm]	178.5 (174.0–180.5)	167.5	192.0	1.8
Body mass [kg]	63.2 (60.1–67.6)	52.6	82.2	5.9
Lean body mass [kg]	53.4 (51.0–57.3)	43.9	67.1	5.9
Fat mass [kg]	9.8 (8.7–12.2)	6.5	15.1	17.9
Fat [%]	15.5 (14.5–17.9)	11.1	19.7	11.0

Q1–Q3—first and third quartiles, CQV—coefficient of quartile variation, TE—training experience.

**Table 2:** Overuse injury survey.

**Question 1** **Have you experienced (location) overuse injury during the past 18 weeks?**			
a. Yes		b. No	
**Question 2** **How many days in total have you had difficulties due to (location) overuse injury during the past 18 weeks?**			
a. 1–3 days	b. 4–7 days	c. 8–28 days	d. >28 days
**Question 3** **To what extent have you reduced your training volume due to (location) overuse injury during the past 18 weeks?**			
a. No reduction	b. To a minor extent	c. To a moderate extent	d. To a major extent
**Question 4** **To what extent has (location) overuse injury affected your performance during the past 18 weeks?**			
a. No effect	b. To a minor extent	c. To a moderate extent	d. To a major extent
**Question 5** **To what extent have you experienced pain related to (location) overuse injury during past 18 weeks?**			
a. To minor extent	b. To moderate extent		c. To major extent

**Table 3:** Score distributions for FMS component tests.

	Score, N (%)			
	0	1	2	3
Deep squat	0 (0.0%)	1 (4.4%)	13 (56.5%)	9 (39.1%)
Hurdle step	0 (0.0%)	7 (30.4%)	16 (69.6%)	0 (0.0%)
In-line lunge	0 (0.0%)	2 (8.7%)	16 (69.6%)	5 (21.7%)
Shoulder mobility	0 (0.0%)	0 (0.0%)	3 (13.0%)	20 (87.0%)
Active straight leg raise	0 (0.0%)	0 (0.0%)	15 (65.2%)	8 (34.8%)
Trunk stability push-up	2 (8.7%)	7 (30.4%)	8 (34.8%)	6 (26.1%)
Rotatory stability	0 (0.0%)	0 (0.0%)	23 (100.0%)	0 (0.0%)

N—number of observations, (%)—percentage of observations.

**Table 4:** Summary of Lower Quarter Y-balance test composite scores.

	Median	Q1–Q3	Min.	Max.	CQV (%)	*p*
YBT-LQ composite score-L (%)	97.9	96.0–101.7	78.0	104.2	2.9	NA
YBT-LQ composite score-R (%)	99.2	95.5–102.8	89.6	108.3	3.7	NA
Difference L vs. R (%)	1.53	0.67–4.05	0.12	6.47	80.5	0.622

Q1–Q3—first and third quartiles, Min.—minimum, Max.—maximum, CQV—coefficient of quartile variation, *p*—probability of Type-1 error.

**Table 5:** Distribution of reach distances (median as well as first and third quartiles) for all directions.

Direction	RD (cm)		Δ (cm)	*p*	RD (%LL)		Δ (%)	*p*
	L	R			L	R		
Anterior	68.2 (63.5–73.8)	69.3 (63.6–73.3)	1.83 (1.00–3.00)	0.888	74.0 (69.3–78.1)	74.0 (70.2–78.2)	2.74 (1.33–4.40)	0.888
Posterolateral	102.0 (95.1–105.3)	103.3 (98.5–106.2)	3.16 (1.25–5.08)	0.324	110.0 (103.5–115.6)	112.4 (106.4–116.7)	3.14 (1.19–5.26)	0.378
Posteromedial	103.2 (100.3–107.3)	105.3 (100.7–109.0)	2.83 (0.92–5.25)	0.630	112.3 (107.2–116.3)	113.3 (108.4–116.6)	2.60 (0.91–5.03)	0.488

RD (cm)—reach distance in centimetres, L, R—left, right leg, (%LL)—reach distance normalised to leg length, Δ—difference between left and right lower limb, *p*—probability of Type-1 error.

**Table 6:** Location and severity of overuse injuries.

Problem Location	(%)	Duration (Days) of Problem (%)		Extent of Training Volume Reduction (%)		Impact on Performance (%)		Extent of Pain Experience (%)	
Knee	26.3	1–3		No reduction		No effect	10.5	Minor	
		4–7	26.3	Minor	10.5	Minor	15.8	Moderate	21.0
		8–28		Moderate	10.5	Moderate		Major	5.3
		>28		Major	5.3	Major			
Hip	0.0	1–3		No reduction		No effect		Minor	
		4–7		Minor		Minor		Moderate	
		8–28		Moderate		Moderate		Major	
		>28		Major		Major			
Back	21.1	1–3	5.3	No reduction	10.5	No effect	5.3	Minor	
		4–7	10.5	Minor	5.3	Minor	10.5	Moderate	10.5
		8–28		Moderate		Moderate		Major	10.5
		>28	5.3	Major	5.3	Major	5.3		
Neck	10.5	1–3		No reduction	10.5	No effect	5.3	Minor	5.3
		4–7	5.3	Minor		Minor	5.3	Moderate	5.3
		8–28		Moderate		Moderate		Major	
		>28	5.3	Major		Major			
Other	5.3	1–3		No reduction		No effect		Minor	
		4–7		Minor		Minor		Moderate	
		8–28	5.3	Moderate		Moderate		Major	5.3
		>28		Major	5.3	Major	5.3		

(%)—percentage of valid observation.

**Table 7:** Indicators of injury risk predictive value for FMS and YBT-LQ.

	Sensitivity (95% CI)	Specificity (95% CI)	Odds Ratio (95% CI)
FMS_CS_ ≤14/21	0.33 (0.04–0.78)	0.62 (0.32–0.86)	0.80 (0.10–6.10)
YBT-LQ_CS-L_ ≤ 94%	0.17 (0.04–0.64)	0.92 (0.64–1.00)	2.40 (0.12–46.39)
YBT-LQ_CS-R_ ≤ 94%	0.33 (0.04–0.78)	0.85 (0.55–0.98)	2.75 (0.28–26.61)
YBT-LQ_A-R-Δ_ ≥ 4 cm	0.17 (0.42–0.64)	0.92 (0.64–1.00)	2.40 (0.12–46.39)
YBT-LQ_PL-R-Δ_ ≥ 4 cm	0.67 (0.22–0.96)	0.69 (0.39–0.91)	4.50 (0.57–35.52)
YBT-LQ_PM-R-Δ_ ≥ 4 cm	0.33 (0.04–0.77)	0.46 (0.19–0.75)	0.43 (0.05–3.22)

FMS_CS_—FMS composite score, YBT-LQ_CS-L,R_—Lower Quarter Y-balance test composite score for left, right lower limb. YBT-LQ_A-R-Δ, PL-R-Δ, PM-R-Δ_—asymmetry in anterior, posterolateral, and posteromedial reach.

## Data Availability

The raw data supporting the conclusions of this article will be made available by the authors, without undue reservation.
